# Reliability and Validity of the Rate of Force Development for Quadriceps in Older Patients with Cardiovascular Disease

**DOI:** 10.3390/jcm13195973

**Published:** 2024-10-08

**Authors:** Takuji Adachi, Chubu Morishima, Yuta Nojiri, Yuki Tsunekawa, Daisuke Tanimura, Taisei Sano, Kenichi Shibata, Hideki Kitamura

**Affiliations:** 1 Department of Integrated Health Sciences, Nagoya University Graduate School of Medicine, Nagoya 461-8673, Japan; 2 Department of Physical Therapy, Nagoya University School of Health Sciences, Nagoya 461-8673, Japan; 3 Department of Rehabilitation, Nagoya Ekisaikai Hospital, Nagoya 454-0854, Japan; 4 Department of Cardiology, Nagoya Ekisaikai Hospital, Nagoya 454-0854, Japan; 5 Department of Cardiac Rehabilitation, Nagoya Heart Center, Nagoya 461-0045, Japan; 6 Department of Cardiovascular Surgery, Nagoya Heart Center, Nagoya 461-0045, Japan

**Keywords:** muscle strength, rate of force development, aging, cardiovascular disease, cardiac rehabilitation

## Abstract

**Background/Objectives**: The rate of force development (RFD), which is the change in force over a period of time during muscle contraction, quantifies rapid muscle contractions. RFD may serve as a measure of physical rehabilitation in patients with cardiovascular disease (CVD); however, its reliability and validity in older patients remain unclear. This study examined the reliability and validity of quadricep RFD in older patients with CVD. **Methods**: This prospective study enrolled 30 outpatients undergoing cardiac rehabilitation (median age, 77 years) and 30 inpatients hospitalized for CVD (median age, 76 years). The quadricep RFD values at three time points (RFD_50_, 0–50 ms; RFD_100_, 0–100 ms; and RFD_200_, 0–200 ms) were calculated from the slope of the force–time curve. Physical performance was assessed using the Short Physical Performance Battery (SPPB). Intra- and inter-rater correlation coefficients were assessed for outpatients. The correlation coefficients between RFD values and physical performance indicators were assessed separately for outpatients and inpatients. **Results**: The intraclass correlation coefficients (1,1) and (2,1) for RFD_50_, RFD_100_, and RFD_200_ were 0.742, 0.893, and 0.873 and 0.810, 0.918, and 0.930, respectively. The correlation coefficients for SPPB with RFD_50_, RFD_100_, and RFD_200_ were 0.553, 0.547, and 0.597 (all *p* < 0.05), respectively, for inpatients; similar moderate correlations were observed for gait speed and the chair stand test. **Conclusions**: The test–retest reliability of the RFD was excellent in older patients with CVD. The RFD was positively correlated with physical function indicators, suggesting its validity as a measure of physical rehabilitation.

## 1. Introduction

Muscular structural integrity and functionality are critical determinants of exercise tolerance and overall quality of life. These factors serve as significant predictors of physical recovery in individuals diagnosed with cardiovascular disease (CVD) or heart failure or those recovering from surgical procedures [1,2]. Guidelines for cardiac rehabilitation and secondary prevention of cardiac events recommend implementing resistance training along with aerobic exercises in exercise-based cardiac rehabilitation programs [3,4]. While the effects of exercise training interventions have predominantly been investigated in younger populations or individuals exhibiting relatively preserved physical function [5,6], the impact of individualized exercise training on older patients with CVD, heart failure, or deteriorating physical function remains underexplored [7].

To implement an effective individualized exercise program, it is essential to understand the functional characteristics and quality of skeletal muscles, as well as the physical limitations of patients [6]. Currently, skeletal muscle assessment in cardiac rehabilitation predominantly emphasizes the evaluation of muscle mass and MVC, reflecting the high prevalence of sarcopenia among patients with CVD or heart failure compared to the general population. Contributory factors for the development of sarcopenia include aging, inadequate nutrition, physical inactivity, and inflammatory states resulting from various underlying diseases, which can lead to diminished anabolic and heightened catabolic processes in the muscles [8]. Ideally, a cardiac rehabilitation program should possess practical relevance and focus on enhancing the functional capacities necessary for daily living by accurately assessing muscle strength in older individuals with CVD and those recovering from surgery.

Assessing instantaneous muscle contraction within 200 milliseconds is a significant predictor of the ability to perform daily activities and prevent falls, whereas a measurement of MVC does not provide predictive value for these activities. The rate of force development (RFD), which is calculated as the change in force over a period of muscle contraction, was used to quantify the rapid force increase [9]. The quantity and functionality of type 2 muscle fibers are important determinants in RFD measurements as well as MCV values. Additionally, early-phase RFD (muscle contraction within 50 or 100 ms) is associated with neurological factors, including motor unit discharge rate, whereas late-phase RFD is influenced by muscle size and muscle–tendon unit stiffness [9], suggesting that RFD measurements may provide deep insights into the causes of decreased physical performance. The RFD has been studied in young athletes and patients with neurological or musculoskeletal diseases; however, there are only a few studies on patients with other chronic diseases [10,11].

RFD values may be more beneficial in designing optimal individualized exercise and postural training programs aimed at enhancing mobility and preventing falls. Therefore, assessment based on RFD values could offer a novel perspective for the rehabilitation of older individuals, potentially proving more effective in mitigating muscle atrophy. However, to the best of our knowledge, the clinical usefulness of the RFD in patients with CVD has not yet been evaluated. RFD measurement reliability has been investigated in younger populations [12,13,14]. As the number of older patients with CVD increases, investigating the reliability of the RFD in this population is clinically significant and serves as a basis for examining its potential usefulness in cardiac rehabilitation. Consequently, this study investigated the reliability and validity of quadricep RFD in older individuals with cardiovascular disease within a clinical context.

## 2. Materials and Methods

### 2.1. Study Design and Participants

This methodological study prospectively enrolled (1) patients aged ≥65 years who participated in an outpatient cardiac rehabilitation program and (2) inpatients aged ≥65 years who were admitted for heart failure or cardiovascular surgery. Consecutive patients who met the eligibility criteria and agreed to participate in the study were enrolled. Participants in the outpatient cardiac rehabilitation program were eligible for inclusion if they had been discharged from the hospital for a minimum of 2 months to avoid the influence of significant changes in physical function and physical activity in the early post-discharge period.

We excluded patients who could not walk, had difficulty understanding the experimental procedures, had neurological disorders, had hemiplegia following stroke, had any orthopedic diseases affecting RFD assessment, or were judged by a physician to be unsuitable for muscle strength measurements for reasons such as circulatory or respiratory status.

### 2.2. Study Procedure

The test–retest reliability of the RFD was evaluated. The RFD assessment comprised three sessions, with each subsequent session occurring one week later to minimize the effects of muscle fatigue on the experimental outcomes and to prevent the overestimation of reliability. Two independent and well-trained raters (Raters 1 and 2) separately measured the RFD of the knee extensor muscles. On the first and second days (after a 1-week interval), the participants were tested using the Rater 1. On the third day (one week after the second day), the participants were tested using Rater 2.

Due to significant differences in disease severity and physical activity levels, the validity of the RFD was analyzed separately for inpatient and outpatient groups. We assessed the association between RFD values measured on the first day and the physical performance of participants in outpatient cardiac rehabilitation. Additionally, we assessed the association between RFD values measured before discharge (commonly 1–2 days before hospital discharge) and the physical performance of inpatients.

### 2.3. RFD Quadricep Measurements

**Figure 1:**
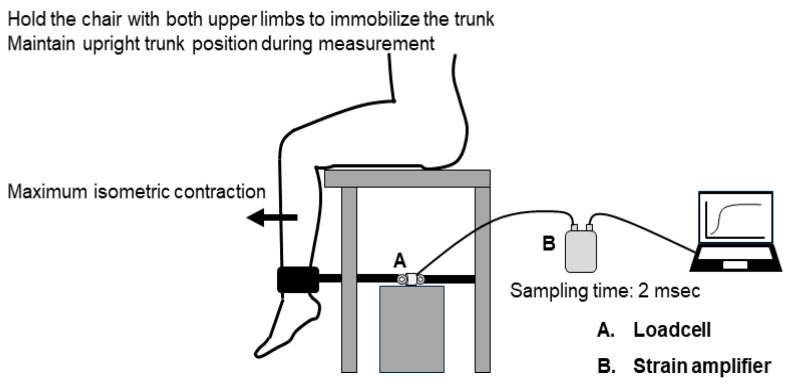
Measurement setup for rate of force development for quadricep isometric strength.

The measurement setup is illustrated in Figure 1. Quadricep isometric strength was measured using a load cell (UNCLB 1KN, UNIPULSE, Tokyo, Japan) fixed to a rigid bar on a bench for measurement (T.K.K.5715a, Takei, Niigata, Japan). As quadricep isometric strength measured in the sitting position can predict exercise capacity and prognosis in patients with CVD [15,16,17], the RFD measurements in this study were also performed in the same position. The participants were seated on a bench with their hips and knees flexed at 90°, ensuring that their toes were not in contact with the floor. A belt attachment was used to connect and fix the load cell to the distal shin.

Participants were instructed to extend their knees for 3 s “as fast with maximum force” as possible following a 3 s countdown [14,18,19,20]. Standardized verbal encouragement was provided by the raters during each trial. The quadricep isometric strength was converted into electrical quantities and subsequently recorded using a strain amplifier (TSA-210, Takei, Niigata, Japan) at a sampling rate of 500 Hz (sampling time of 2 ms). The strain amplifier device was connected to a personal computer via a universal serial bus (USB) cable. The rater confirmed that each measurement trial was properly performed by checking the muscle force waveform displayed on the monitor. Three trials were performed for each leg after each practice trial. The rest time between trials was set at 2 min. The moment arm was measured from the lateral knee joint space to the median belt space.

RFD values were calculated as the mean change in torque per second (delta torque/delta time) during the intervals of 0–50 ms (RFD_50_), 0–100 ms (RFD_100_), and 0–200 ms (RFD_200_) [21]. The MVC values were then calculated. All muscle force data analyses were performed using MATLAB (MathWorks, Natick, MA, USA). Torque onset was identified using the “findchangepts” function of the MATLAB software version 24.2, which returns the index at which the mean of the parameter changes most significantly. The baseline value was calculated using the 1 s mean value before the onset of the identified torque. Based on previous studies [9,22], a zero-phase low-pass digital filter (fourth-order Butterworth, cut-off frequency: 14 Hz) was used to remove noise in the high-frequency components and prevent time shifts when using a normal smoothing function. RFD values and MVC were divided by body weight (units: Nm/kg/s for RFDs; Nm/kg for MVC), and the highest values were used for the analyses.

### 2.4. Physical Performance Test

The Short Physical Performance Battery (SPPB) was used to evaluate lower-limb physical performance. We measured SPPB to evaluate the validity of the RFD in order to analyze the relationship between gait and postural control ability rather than aerobic capacity or symptom severity. The SPPB consists of three physical performance tests related to mobility: the 4 m walk time, five repeated chair stands, and static standing balance [23]. Each test was scored from 1 (worst) to 4 (best performance) based on the quartile distribution of the test results in an older reference population. A score of 0 indicated that the patient was unable to complete the individual test. For gait speed assessment, the participants’ usual speed was measured during a 4 m walk. In the chair stand test, participants were instructed to stand and sit five times as quickly as possible. For the balance tests, the participants were instructed to maintain their feet in side-by-side, semi-tandem, and tandem positions for 10 s/position. The SPPB score (range: 0–12) is the sum of the individual test scores, with higher scores indicating better performance. The SPPB score is a reliable [24] and widely used physical performance test for patients with CVD. The SPPB score can predict exercise tolerance and prognosis in patients with CVD [25,26]. Additionally, it has been reported that this measure predicts the incidence of CVD in community-dwelling older individuals without a known history of CVD [27]. Based on these previous findings, SPPB is a good proxy for cardiac status and has recently been used in clinical trials [28]. An SPPB score of ≤9 points has been used as a cut-off for decreased physical performance [29]. In Japanese adults, a ceiling effect has often been observed in this test, with a high proportion having a score of 11 or 12 points, even among patients with CVD [30]. Therefore, the 4 m walk (gait speed) and chair stand tests (time required for completion), which can be evaluated as continuous variables, were used to assess RFD validity.

### 2.5. Clinical Data

Participant medical records were reviewed to collect data regarding age, sex, height, etiology, comorbidities, left ventricular ejection fraction, biochemical data, and prescribed medications. Comorbidities were defined based on diagnostic or treatment history. Body mass index was calculated using body weight on the day the RFD measurement was performed.

### 2.6. Statistical Analyses

Continuous variables are expressed as the mean and standard deviation for normally distributed variables and as the median with the interquartile range (IQR) for non-normally distributed data. Normality was assessed using the Shapiro–Wilk test and histograms. Categorical data are expressed as numbers and percentages. Characteristics were compared between outpatients and inpatients using the unpaired *t*-test, Mann–Whitney U test, and chi-square test, as appropriate. Pearson’s correlation coefficients for MVC with RFD_50_, RFD_100_, and RFD_200_ were calculated separately for outpatients and inpatients.

The intra- and inter-rater reliabilities of the RFD and MVC were evaluated using the intraclass correlation coefficient (ICC) with a 95% confidence interval (CI) among outpatients who participated in cardiac rehabilitation. Intra-rater reliability was evaluated using ICC (1,1), and inter-rater reliability was determined using ICC (2,1). ICC values were interpreted as follows: <0.5, poor reliability; 0.5–0.75, moderate reliability; 0.75–0.90, good reliability; and >0.90, excellent reliability [31]. RFD validity was examined by calculating Spearman’s correlation coefficient (correlation with SPPB) and Pearson’s correlation coefficient (correlation with gait speed and chair stand test). SPPB scores were reported to show a left-skewed distribution in Japanese patients [30], and Spearman’s correlation coefficient was used. Correlation coefficients were calculated for both inpatients and outpatients.

All statistical analyses were performed using Stata/SE software (version 15.1; StataCorp LP, College Station, TX, USA). Results were considered statistically significant at *p* < 0.05.

### 2.7. Ethics

This study was performed in accordance with the principles of the Declaration of Helsinki and the Japanese Ethical Guidelines for Medical and Biological Research Involving Human Subjects. The study protocol was approved by the Ethics Committee of Nagoya University School of Medicine (15 September 2023; approval number: 2023–0223) and the Research Ethics Committee of the School of Health Sciences, Nagoya University (11 May 2023; approval number: 22–523). All participants provided written informed consent.

## 3. Results

**Figure 2:**
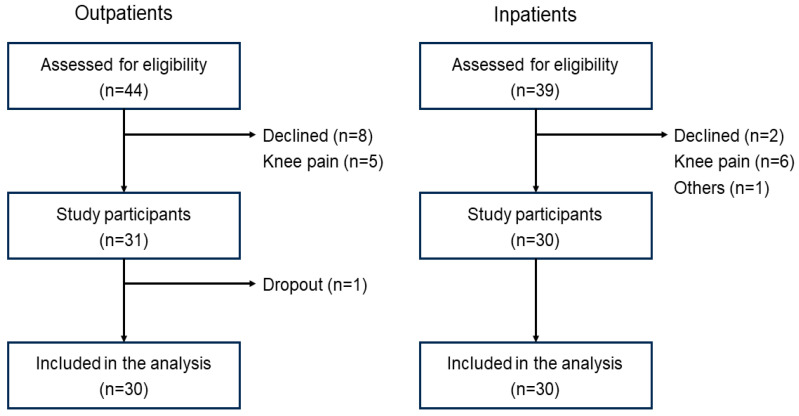
Patient selection flowchart.

A flowchart of the patient selection process is shown in Figure 2. The characteristics of the study participants are presented in Table 1. The median ages of the outpatients (*n* = 30) and inpatients (*n* = 30) were 77 (IQR 72–81) years and 76 (IQR 74–82) years, respectively. None of the outpatients had an SPPB score of ≤9 points. The prevalence of inpatients with an SPPB score of 9 points at discharge was 56.7% (*n* = 17).

MVC had significant correlations with the RFD values of outpatients (RFD_50_, r = 0.486, *p* = 0.007; RFD_100_, r = 0.594, *p* = 0.001; RFD_200_, r = 0.705, *p* < 0.001) and inpatients (RFD_50_, r = 0.494, *p* = 0.006; RFD_100_, r = 0.631, *p* < 0.001; RFD_200_, r = 0.743, *p* < 0.001).

The inter-rater reliability results are presented in Table 2. The ICCs (1,1) of RFD_50_, RFD_100_, RFD_200_, and MVC were 0.742 [95% CI 0.523–0.868], 0.893 [95% CI 0.528–0.963], 0.873 [95% CI 0.636–0.948], and 0.943 [95% CI 0.886–0.973], respectively. The intra-rater reliability results are presented in Table 2. The ICCs (1,2) of RFD_50_, RFD_100_, RFD_200_, and MVC were 0.810 [95% CI 0.640–0.904], 0.918 [95% CI 0.837–0.961], 0.930 [95% CI 0.858–0.966], and 0.947 [95% CI 0.891–0.974], respectively.

**Figure 3:**
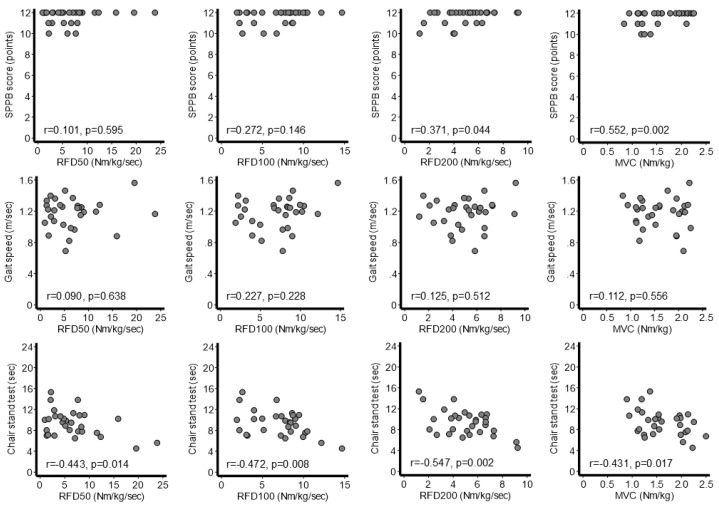
Correlations between physical performance and muscle strength indicators in outpatients (*n* = 30). Muscle strength indicators measured on the first day were analyzed. MVC, maximal voluntary contraction; RFD, rate of force development; SPPB, Short Physical Performance Battery.

The correlations among RFD values, MVC, and physical performance in outpatients are shown in Figure 3. SPPB was significantly associated with RFD_200_ (r = 0.371, *p* = 0.044) and MVC (r = 0.552, *p* = 0.002), although not with RFD_50_ or RFD_100_. The 4 m gait speed was not significantly associated with any muscle strength parameters. The chair stand test time was significantly associated with RFD_50_ (r = −0.443, *p* = 0.014), RFD_100_ (r = −0.472, *p* = 0.008), RFD_200_ (r = −0.547, *p* = 0.002), and MVC (r = −0.431, *p* = 0.017).

**Figure 4:**
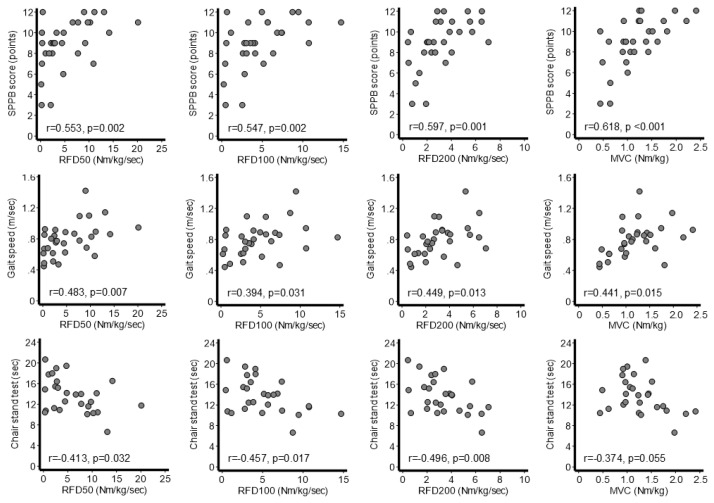
Correlations between physical performance and muscle strength indicators in inpatients (*n* = 30). MVC, maximal voluntary contraction; RFD, rate of force development; SPPB, Short Physical Performance Battery.

The correlations among the RFD values, MVC, and physical performance at discharge among inpatients are shown in Figure 4. Spearman’s correlation coefficients between SPPB and RFD_50_, RFD_100_, RFD_200_, and MVC were 0.553, 0.547, 0.597, and 0.618 (*p* < 0.05 for all), respectively. Pearson’s correlation coefficients between the 4 m gait speed and RFD_50_, RFD_100_, RFD_200_, and MVC were 0.483, 0.394, 0.449, and 0.441, respectively (*p* < 0.05 for all). Pearson’s correlation coefficients between the chair stand test time and RFD_50_, RFD_100_, RFD_200_, and MVC were −0.413, −0.457, −0.496 (*p* < 0.05 for all), and −0.374 (*p* = 0.055), respectively.

## 4. Discussion

This study investigated the reliability and validity of RFD measurements in older patients with CVD. Overall, the RFD values demonstrated excellent test–retest reliability. The RFD was correlated with physical performance indicators, suggesting its validity as a measure of physical rehabilitation in older patients with CVD. The RFD and MVC had a significant positive correlation, although the correlation coefficient was smaller for RFD at an earlier time after the force onset. This suggests that early RFD may reflect different aspects of muscle strength compared to MVC, which is a widely used conventional muscle strength measure. Although the functional limitations in patients with various CVDs cannot be determined solely by muscle strength, the findings from this study may provide preliminary data to support the incorporation of RFD assessment in the formulation of optimal exercise training programs aimed at enhancing functional mobility in older individuals.

The test–retest reliability of RFD measurements among older patients in this study was comparable to that in a healthy younger population in previous studies. RFD measurements were studied using large laboratory-based measuring instruments; however, it was shown that even simple measurements using a handheld dynamometer have good reproducibility [12,13,14]. The present study supports these findings and demonstrates the feasibility of RFD measurements in clinical settings when resources and space are limited. Careful interpretation of our study results is essential for patients who may have experienced significant changes in health status during hospitalization or for those in the inpatient setting. This caution is particularly relevant as our study focused on individuals in the outpatient setting who maintained good physical function. This was because the short length of hospital stay, in addition to the dramatic change in health status during hospitalization, limited the examination of the reliability of the RFD among inpatients. The measurement procedures and instructions should be well controlled, and patient understanding of the task, especially for those with cognitive decline, should be confirmed by pre-practice sessions. Nevertheless, this study has clinical significance because it provides fundamental data for examining the usefulness of the RFD in older patients with chronic diseases.

There was a moderate correlation between the RFD and physical performance, suggesting the validity of the RFD as a measure of physical rehabilitation in older patients with CVD. Lower-extremity RFD reportedly decreases for various reasons, including aging [9], knee osteoarthritis [32], neurodegenerative diseases [33], and cerebrovascular diseases [34,35,36]. Instantaneous muscle contractions, including the RFD, are predictive of physical performance [37], frailty [38], and mobility [39] in community-dwelling older adults. A systematic review of neurodegenerative diseases suggested that lower-extremity RFD is sensitive to neurodegeneration and could serve as a useful indicator of changes in neuromuscular function elicited by neurodegeneration. However, evidence for the clinical usefulness of the RFD in patients with CVD is lacking. A previous report showed a positive association between quadricep RFD and sit-to-stand performance in patients with chronic kidney disease [10]. Another study demonstrated that patients with chronic kidney disease have higher fatigability than controls, which may be associated with impaired motor unit recruitment [11]. In this study, the RFD was correlated with SPPB score, gait speed, and chair stand test performance in patients hospitalized for heart failure or cardiovascular surgery. The results of this study imply that the association between the RFD and physical function may vary depending on the type of physical function indicator and the RFD time interval. Larger studies are needed for more detailed analyses. Additionally, the impact of sex on the study findings should be considered because of the difference in cross-sectional areas of type 2 fibers between men and women [40].

The correlation between the RFD and the SPPB score was higher in inpatients than in outpatients. In this study, outpatients exhibited high SPPB scores (median, 12; IQR, 11–12), which may have contributed to a low correlation coefficient between the RFD and SPPB. Conversely, among hospitalized patients, the inclusion of individuals with lower physical function likely resulted in a broader distribution of SPPB scores and a stronger correlation with the RFD. Additionally, the non-linear relationship between leg muscle strength and gait speed in older populations [41] may elucidate these findings, as outpatients demonstrated superior muscle strength and physical function compared to inpatients.

In this study, earlier RFD values had smaller correlation coefficients with MVC. These results are consistent with a previous study of healthy younger men, which reported correlation coefficients of RFD_50_, RFD_100_, and RFD_200_ with MVC of approximately 0.5, 0.6, and 0.8, respectively [42]. Early- (≤100 ms) and late-phase (≥200 ms) RFD values reportedly have different determinants. Early RFD values appear to be affected by neurological factors, as indicated by their relationship with electromyography activity [9,42,43]. These findings suggest that exercise and postural control training may improve physical performance without mediating changes in muscle mass. A previous study on resistance-type training reported improvements in muscle strength and physical performance in older individuals without an increase in muscle mass [44]. A recent randomized controlled trial demonstrated that tailored physical rehabilitation improved the physical performance and quality of life of older patients with heart failure, and the effects were greater for patients with than without frailty [28,45]. Although exercise training and nutritional support focused on increasing muscle mass are recommended for patients with impaired physical function, individualized training to improve muscle contraction efficiency may be a novel intervention to improve physical performance.

Due to the preliminary nature of this study, there are insufficient data regarding the clinical utility of the RFD in cardiac rehabilitation. The mechanisms underlying the decline in physical function and exercise tolerance are multifactorial, encompassing factors such as aging, cardiovascular function, comorbidities, and energy metabolism [46]. These elements should be analyzed to investigate the causes of the decline in the RFD. Additionally, comparing the responsiveness of the RFD to exercise and treatment against conventional muscle strength parameters may present a valuable avenue for further research. Despite the limited evidence available in older populations, movement velocity during resistance training may significantly influence training outcomes related to muscle size, MVC, and RFD [47,48]. Finally, this study examined only the preliminary associations between the RFD and the SPPB and its subcomponents. The relationship between the RFD and other indicators of physical function, including physical frailty and body composition, warrants further investigation.

This study had several limitations. First, the generalizability of our results should be carefully considered owing to the small sample size. Second, subgroup sensitivity analysis for different patient characteristics could not be performed because of the limited size of the study population. As the patients analyzed in this study were hospitalized because of heart failure or cardiovascular surgery, a detailed analysis should be performed for a larger number of cases. Third, although we demonstrated the reproducibility and validity of quadricep RFD measured in a simple setting, the methodology was primarily designed for research purposes. The availability of the necessary devices may pose a challenge for routine clinical practice. Fourth, several potential confounding factors, such as disease severity, cognitive function, and physical activity, may have affected the relationship between the RFD and physical performance. Therefore, the results of this study should be considered as preliminary data for hypothesis generation. Finally, because this was a cross-sectional analysis, the longitudinal association between the RFD and functional prognosis was not evaluated.

In conclusion, the test–retest reliability of the RFD was excellent in older patients with CVD, even with a simple measurement setup. The RFD was positively correlated with SPPB score, gait speed, and chair stand test performance. The RFD reflects distinct aspects of muscle force compared to MVC and is recognized as an index closely associated with ambulatory capacity and daily living activities. Although this study was preliminary in nature, the evaluation of RFD may contribute to the development of individualized strength training protocols based on a patient’s muscle strength and physical function in elderly individuals with CVD.

## Figures and Tables

**Table 1:** Characteristics of the study participants.

	Outpatients (*n* = 30)	Inpatients (*n* = 30)	*p*
Age, years	77 (72–81)	76 (74–82)	0.689
Men	70.0%	36.7%	0.010
Body mass index, kg/m^2^	22.2 ± 3.1	21.8 ± 4.1	0.691
Heart failure	50.0%	33.3%	0.190
Cardiovascular surgery			
CABG	13.3%	3.3%	0.161
Valvular surgery	33.3%	30.0%	0.781
Aortic surgery	3.3%	16.7%	0.071
Concomitant surgery	6.7%	23.3%	0.085
Hypertension	53.3%	49.8%	0.602
Dyslipidemia	63.3%	53.3%	0.432
Diabetes mellitus	50.0%	40.7%	0.015
Left ventricular ejection fraction, %	51 (41–60)	55 (45–61)	0.085
NT-proBNP, pg/mL	345 (151–792)	1126 (353–1745)	0.003
MMSE, points	28 (26–30)	27 (23–29)	0.065
SPPB, points	12 (11–12)	9 (8–11)	<0.001

Continuous variables are expressed as mean ± standard deviation or median (interquartile range). CABG, coronary artery bypass grafting; BNP, N-terminal pro-brain natriuretic peptide; MMSE, Mini-Mental State Examination; SPPB, Short Physical Performance Battery.

**Table 2:** Results of inter-rater and intra-rater reliabilities in outpatients (*n* = 30).

	Intra-Rater Reliability			Inter-Rater Reliability		
	Mean ± SD		ICC (1,1) [95% CI]	Mean ± SD		ICC (2,1) [95% CI]
	Day 1	Day 2		Rater 1	Rater 2	
RFD_50_ (Nm/kg/s)	6.76 ± 5.45	6.86 ± 6.01	0.742 [0.523–0.868]	6.86 ± 6.01	6.37 ± 4.84	0.810 [0.640–0.904]
RFD_100_ (Nm/kg/s)	7.07 ± 3.18	6.08 ± 3.09	0.893 [0.528–0.963]	6.08 ± 3.09	6.14 ± 2.87	0.918 [0.837–0.961]
RFD_200_ (Nm/kg/s)	5.07 ± 2.01	4.48 ± 2.04	0.873 [0.636–0.948]	4.48 ± 2.04	4.52 ± 1.82	0.930 [0.858–0.966]
MVC (Nm/kg)	1.62 ± 0.44	1.56 ± 0.42	0.943 [0.886–0.973]	1.56 ± 0.42	1.55 ± 0.37	0.947 [0.891–0.974]

SD, standard deviation; ICC, inter- or intra-rater correlation coefficient; CI, confidence interval; RFD, rate of force development; MVC, maximal voluntary contraction. The ICC (1,1) was calculated based on the two measurements (Day 1 and Day 2) by Rater 1. The ICC (2,1) was calculated based on the measurements of Rater 1 on Day 2 and Rater 2.

## Data Availability

The data presented in this study will be available on reasonable request from the corresponding author due to the privacy of the individuals and ethical reasons.

## References

[1] Carbone S., Kirkman D.L., Garten R.S., Rodriguez-Miguelez P., Artero E.G., Lee D.C., Lavie C.J. (2020). Muscular strength and cardiovascular disease: An updated state-of-the-art narrative review. J. Cardiopulm. Rehabil. Prev..

[2] Kirkman D.L., Lee D.-C., Carbone S. (2022). Resistance exercise for cardiac rehabilitation. Prog. Cardiovasc. Dis..

[3] Abreu A., Frederix I., Dendale P., Janssen A., Doherty P., Piepoli M.F., Völler H., Davos C.H., Ambrosetti M. (2021). Standardization and quality improvement of secondary prevention through cardiovascular rehabilitation programmes in Europe: The avenue towards EAPC accreditation programme: A position statement of the secondary prevention and rehabilitation section of the European Association of Preventive Cardiology (EAPC). Eur. J. Prev. Cardiol..

[4] Makita S., Yasu T., Akashi Y.J., Adachi H., Izawa H., Ishihara S., Iso Y., Ohuchi H., Omiya K., Ohya Y. (2022). JCS/JACR 2021 guideline on rehabilitation in patients with cardiovascular disease. Cir. J..

[5] Prokopidis K., Isanejad M., Akpan A., Stefil M., Tajik B., Giannos P., Venturelli M., Sankaranarayanan R. (2022). Exercise and nutritional interventions on sarcopenia and frailty in heart failure: A narrative review of systematic reviews and meta-analyses. ESC Heart Fail..

[6] Bjarnason-Wehrens B., Schwaab B., Reiss N., Schmidt T. (2022). Resistance training in patients with coronary artery disease, heart failure, and valvular heart disease. J. Cardiopulm. Rehabil. Prev..

[7] McDonagh T.A., Metra M., Adamo M., Gardner R.S., Baumbach A., Böhm M., Burri H., Butler J., Celutkiene J., Chioncel O. (2021). 2021 ESC guidelines for the diagnosis and treatment of acute and chronic heart failure. Eur. Heart J..

[8] Zuo X., Li X., Tang K., Zhao R., Wu M., Wang Y., Li T. (2023). Sarcopenia and cardiovascular diseases: A systematic review and meta-analysis. J. Cachexia Sarcopenia Muscle.

[9] Maffiuletti N.A., Aagaard P., Blazevich A.J., Folland J., Tillin N., Duchateau J. (2016). Rate of force development: Physiological and methodological considerations. Eur. J. Appl. Physiol..

[10] Gollie J.M., Harris-Love M.O., Patel S.S., Shara N.M., Blackman M.R. (2021). Rate of force development is related to maximal force and sit-to-stand performance in men with stages 3b and 4 chronic kidney disease. Front. Rehabil. Sci..

[11] Chatrenet A., Piccoli G., Audebrand J.M., Torreggiani M., Barbieux J., Vaillant C., Morel B., Durand S., Beaune B. (2023). Analysis of the rate of force development reveals high neuromuscular fatigability in elderly patients with chronic kidney disease. J. Cachexia Sarcopenia Muscle.

[12] Mentiplay B.F., Perraton L.G., Bower K.J., Adair B., Pua Y.-H., Williams G.P., McGaw R., Clark R.A. (2015). Assessment of lower limb muscle strength and power using hand-held and fixed dynamometry: A reliability and validity study. PLoS ONE.

[13] Takeda K., Tanabe S., Koyama S., Nagai T., Sakurai H., Kanada Y., Shomoto K. (2018). Intra- and inter-rater reliability of the rate of force development of hip abductor muscles measured by hand-held dynamometer. Meas. Phys. Educ. Exerc. Sci..

[14] Ishøi L., Hölmich P., Thorborg K. (2019). Measures of hip muscle strength and rate of force development using a fixated handheld dynamometer: Intra-tester intra-day reliability of a clinical set-up. Int. J. Sports Phys. Ther..

[15] Kamiya K., Mezzani A., Hotta K., Shimizu R., Kamekawa D., Noda C., Yamaoka-Tojo M., Matsunaga A., Masuda T. (2014). Quadriceps isometric strength as a predictor of exercise capacity in coronary artery disease patients. Eur. J. Prev. Cardiol..

[16] Kamiya K., Masuda T., Tanaka S., Hamazaki N., Matsue Y., Mezzani A., Matsuzawa R., Nozaki K., Maekawa E., Noda C. (2015). Quadriceps strength as a predictor of mortality in coronary artery disease. Am. J. Med..

[17] Nakamura T., Kamiya K., Hamazaki N., Matsuzawa R., Nozaki K., Ichikawa T., Yamashita M., Maekawa E., Reed J.L., Noda C. (2021). Quadriceps strength and mortality in older patients with heart failure. Can. J. Cardiol..

[18] Sahaly R., Vandewalle H., Driss T., Monod H. (2001). Maximal voluntary force and rate of force development in humans—Importance of instruction. Eur. J. Appl. Physiol..

[19] Jaafar H., Lajili H. (2018). The influence of verbal instruction on measurement reliability and explosive neuromuscular performance of the knee extensors. J. Hum. Kinet..

[20] Moir G.L., Getz A., Davis S.E., Marques M., Witmer C.A. (2019). The inter-session reliability of isometric force-time variables and the effects of filtering and starting force. J. Hum. Kinet..

[21] Aagaard P., Simonsen E.B., Andersen J.L., Magnusson P., Dyhre-Poulsen P. (2002). Increased rate of force development and neural drive of human skeletal muscle following resistance training. J. Appl. Physiol..

[22] Blazevich A.J., Horne S., Cannavan D., Coleman D.R., Aagaard P. (2008). Effect of contraction mode of slow-speed resistance training on the maximum rate of force development in the human quadriceps. Muscle Nerve.

[23] Guralnik J.M., Simonsick E.M., Ferrucci L., Glynn R.J., Berkman L.F., Blazer D.G., Scherr P.A., Wallace R.B. (1994). A short physical performance battery assessing lower extremity function: Association with self-reported disability and prediction of mortality and nursing home admission. J. Gerontol..

[24] Ostir G.V., Volpato S., Fried L.P., Chaves P., Guralnik J.M. (2002). Reliability and sensitivity to change assessed for a summary measure of lower body function: Results from the women’s health and aging study. J. Clin. Epidemiol..

[25] Kitai T., Shimogai T., Tang W.H.W., Iwata K., Xanthopoulos A., Otsuka S., Nakada F., Yokoyama R., Kamiya K., Saito H. (2021). Short physical performance battery vs. 6-minute walking test in hospitalized elderly patients with heart failure. Eur. Heart J. Open.

[26] Shibata K., Kameshima M., Adachi T., Kito H., Tanaka C., Sano T., Tanaka M., Suzuki Y., Tamaki M., Kitamura H. (2024). Association between preoperative phase angle and all-cause mortality after cardiovascular surgery: A retrospective cohort study. J. Cachexia Sarcopenia Muscle.

[27] Bellettiere J., Lamonte M.J., Unkart J., Liles S., Laddu-Patel D., Manson J.E., Banack H., Seguin-Fowler R., Chavez P., Tinker L.F. (2020). Short physical performance battery and incident cardiovascular events among older women. J. Am. Heart Assoc..

[28] Kitzman D.W., Whellan D.J., Duncan P., Pastva A.M., Mentz R.J., Reeves G.R., Nelson M.B., Chen H., Upadhya B., Reed S.D. (2021). Physical rehabilitation for older patients hospitalized for heart failure. New Engl. J. Med..

[29] Chen L.K., Woo J., Assantachai P., Auyeung T.W., Chou M.Y., Iijima K., Jang H.C., Kang L., Kim M., Kim S. (2020). Asian working group for sarcopenia: 2019 consensus update on sarcopenia diagnosis and treatment. J. Am. Med. Dir. Assoc..

[30] Ogawa M., Satomi-Kobayashi S., Yoshida N., Tsuboi Y., Komaki K., Nanba N., Izawa K.P., Sakai Y., Akashi M., Hirata K. (2021). Relationship between oral health and physical frailty in patients with cardiovascular disease. J. Cardiol..

[31] Koo T.K., Li M.Y. (2016). A Guideline of selecting and reporting intraclass correlation coefficients for reliability research. J. Chiropr. Med..

[32] Suzuki Y., Iijima H., Nakamura M., Aoyama T. (2022). Rate of force development in the quadriceps of individuals with severe knee osteoarthritis: A preliminary cross-sectional study. PLoS ONE.

[33] Lomborg S.D., Dalgas U., Hvid L.G. (2022). The importance of neuromuscular rate of force development for physical function in aging and common neurodegenerative disorders—A systematic review. J. Musculoskelet. Neuronal Interact..

[34] Horstman A.M., Gerrits K.H., Beltman M.J., Koppe P.A., Janssen T.W., de Haan A. (2010). Intrinsic properties of the knee extensor muscles after subacute stroke. Arch. Phys. Med. Rehabil..

[35] Fimland M.S., Moen P.M.R., Hill T., Gjellesvik T.I., Tørhaug T., Helgerud J., Hoff J. (2011). Neuromuscular performance of paretic versus non-paretic plantar flexors after stroke. Eur. J. Appl. Physiol..

[36] Shimose R., Shimizu S., Onodera A., Shibata K., Ichinosawa Y., Enoki I., Tsunoda S., Miura H., Watanabe H., Matsunaga A. (2019). Decreased rate of leg extensor force development in independently ambulant patients with acute stroke with mild paresis. J. Biomech..

[37] Osawa Y., Studenski S.A., Ferrucci L. (2018). Knee extension rate of torque development and peak torque: Associations with lower extremity function. J. Cachexia Sarcopenia Muscle.

[38] Burbank C.M., Branscum A., Bovbjerg M.L., Hooker K., Smit E. (2023). Muscle Power Predicts Frailty Status over Four Years: A Retrospective Cohort Study of the National Health and Aging Trends Study. J. Frailty Sarcopenia Falls.

[39] Hester G.M., Ha P.L., Dalton B.E., Vandusseldorp T.A., Olmos A.A., Stratton M.T., Bailly A.R., Vroman T.M. (2021). Rate of force development as a predictor of mobility in community-dwelling older adults. J. Geriatr. Phys. Ther..

[40] Staron R.S., Hagerman F.C., Hikida R.S., Murray T.F., Hostler D.P., Crill M.T., Ragg K.E., Toma K. (2000). Fiber type composition of the vastus lateralis muscle of young men and women. J. Histochem. Cytochem..

[41] Buchner D.M., Larson E.B., Wagner E.H., Koepsell T.D., de Lateur B.J. (1996). Evidence for a non-linear relationship between leg strength and gait speed. Age Ageing.

[42] Cossich V., Maffiuletti N.A. (2020). Early vs. late rate of torque development: Relation with maximal strength and influencing factors. J. Electromyogr. Kinesiol..

[43] Quinlan J.I., Maganaris C.N., Franchi M.V., Smith K., Atherton P.J., Szewczyk N.J., Greenhaff P.L., Phillips B.E., Blackwell J.I., Boereboom C. (2018). Muscle and tendon contributions to reduced rate of torque development in healthy older males. J. Gerontol..

[44] Churchward-Venne T.A., Tieland M., Verdijk L.B., Leenders M., Dirks M.L., de Groot L.C.P.G.M., van Loon L.J.C. (2015). There are no nonresponders to resistance-type exercise training in older men and women. J. Am. Med. Dir. Assoc..

[45] Pandey A., Kitzman D.W., Nelson M.B., Pastva A.M., Duncan P., Whellan D.J., Mentz R.J., Chen H., Upadhya B., Reeves G.R. (2023). Frailty and effects of a multidomain physical rehabilitation intervention among older patients hospitalized for acute heart failure: A secondary analysis of a randomized clinical trial. JAMA Cardiol..

[46] Mann N., Rosenzweig A. (2012). Can exercise teach us how to treat heart disease?. Circulation.

[47] Claflin D.R., Larkin L.M., Cederna P.S., Horowitz J.F., Alexander N.B., Cole N.M., Galecki A.T., Chen S., Nyquist L.V., Carlson B.M. (2011). Effects of high- and low-velocity resistance training on the contractile properties of skeletal muscle fibers from young and older humans. J. Appl. Physiol..

[48] Guizelini P.C., de Aguiar R.A., Denadai B.S., Caputo F., Greco C.C. (2018). Effect of resistance training on muscle strength and rate of force development in healthy older adults: A systematic review and meta-analysis. Exp. Gerontol..

